# Prediction of solution properties and dynamics of RNAs by means of Brownian dynamics simulation of coarse-grained models: Ribosomal 5S RNA and phenylalanine transfer RNA

**DOI:** 10.1186/s13628-015-0025-7

**Published:** 2015-12-01

**Authors:** Aarón Ayllón Benítez, José Ginés Hernández Cifre, Francisco Guillermo Díaz Baños, José García de la Torre

**Affiliations:** Departamento de Química Física, Universidad de Murcia, Murcia, 30100 Spain

**Keywords:** Brownian dynamics, Coarse-grained model, Hydrodynamics, Transfer RNA, Ribosomal RNA, Diffusion coefficients, Internal dynamics

## Abstract

**Background:**

The possibility of validating biological macromolecules with locally disordered domains like RNA against solution properties is helpful to understand their function. In this work, we present a computational scheme for predicting global properties and mimicking the internal dynamics of RNA molecules in solution. A simple coarse-grained model with one bead per nucleotide and two types of intra-molecular interactions (elastic interactions and excluded volume interactions) is used to represent the RNA chain. The elastic interactions are modeled by a set of Hooke springs that form a minimalist elastic network. The Brownian dynamics technique is employed to simulate the time evolution of the RNA conformations.

**Results:**

That scheme is applied to the 5S ribosomal RNA of *E. Coli* and the yeast phenylalanine transfer RNA. From the Brownian trajectory, several solution properties (radius of gyration, translational diffusion coefficient, and a rotational relaxation time) are calculated. For the case of yeast phenylalanine transfer RNA, the time evolution and the probability distribution of the inter-arm angle is also computed.

**Conclusions:**

The general good agreement between our results and some experimental data indicates that the model is able to capture the tertiary structure of RNA in solution. Our simulation results also compare quite well with other numerical data. An advantage of the scheme described here is the possibility of visualizing the real time macromolecular dynamics.

**Electronic supplementary material:**

The online version of this article (doi:10.1186/s13628-015-0025-7) contains supplementary material, which is available to authorized users.

## Background

Ribonucleic acid, or RNA, is a key molecule for the synthesis of proteins in living cells. Transfer RNA (tRNA) carries an amino acid to the protein synthetic machinery of a cell, the ribosome, which is the particle responsible for translation of the genetic code. Ribosomes are mainly formed by the so-called ribosomal RNA (rRNA) along with a variety of protein subunits. As many biological macromolecules, the RNA functionality is related with its structure which, in turn, determines the RNA solution properties. Therefore, the latter are essential sources of information on RNA structure and dynamics [1]. However, the structural determination of even small RNA molecules like tRNAs (70 to 80 nucleotides long) or the 5S rRNA of *E. Coli* (120 nucleotides long [2]) is not an easy task, being most RNAs much larger molecules. For instance, besides 5S rRNA, the main classes of rRNA are 16S and 23S in prokaryotic cells, and 17S, 18S, 26S, and 28S in eukaryotic cells [2] (S stands for Svedberg, the unit of sedimentation coefficient; it is noteworthy that the notation for ribosomal RNAs, as for some other biomacromolecules, is made in terms of a hydrodynamic property).

**Fig. 1:**
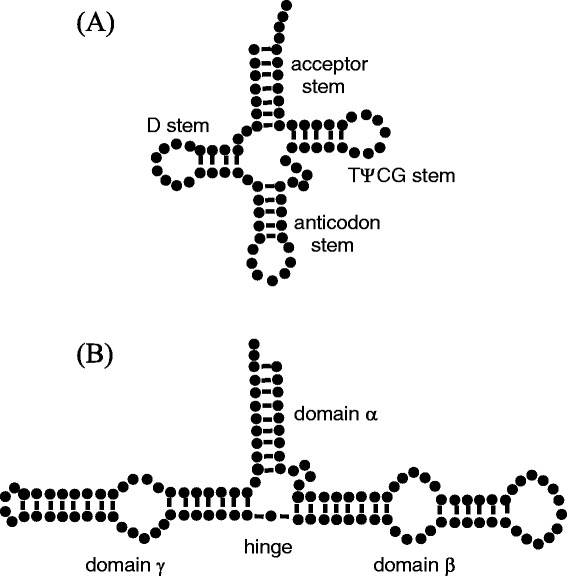
Secondary structure of RNAs. **a** Sketch of the secondary structure of yeast tRNA ^phe^ [45] (note the presence of four helices, three loops and a hinge). **b** Sketch of the secondary structure of the 5S rRNA [20] (note the presence of five double helices, four loops, and a hinge). In each case nucleotides are the black points, double helices are the regions with connections between opposite nucleotides and loops are the “circular” regions without connections between opposite nucleotides

It is well-known that tRNA and rRNA are single-stranded chains that fold unto themselves giving rise to a secondary structure that contains both ordered (essentially rigid) double-helical regions and disordered zones so-called loops (see Fig. 1). Double helices are formed when complementary base pairs belonging to two pieces of the strand interact with each other – after strand folding – through either Watson-Crick or Hoogsteen (non-Watson-Crick) interactions, whereas loops appear in regions with unpaired bases. The overall view of such structure can be described as some number of arms, or stems, joined by the loop regions, which may act as flexible hinges. Thus, RNA molecules have an intrinsic flexibility that allows them to adopt a wide range of three-dimensional conformations. Although valuable insights about the tertiary structure can be obtained from atomic coordinates coming from x-ray crystallography measurements – available at the Protein Data Bank (PDB) (www.rcsb.org) – [3], experiments where the RNA flexibility can be revealed are most useful to characterize its conformational variability. In that sense, as above mentioned, dilute solution properties (hydrodynamic coefficients, scattering-related quantities, etc.) provide key information on the overall three-dimensional structure of RNA when combined with theoretical predictive models. Clearly, those models should include flexibility in order to make adequate predictions.

The tRNA is a particularly simple case as it presents four short stems as sketched in Fig. 1a. In usual conditions, those stems appear grouped forming two arms joined by a disordered region so that the crystal structure has the typical “L-letter” shape [4]. Thus, tRNA has been presented as a typical case among segmentally flexible macromolecules, composed by rigid parts connected by flexible hinges. Considerable effort has been devoted to the characterization of the structure and degree of flexibility of tRNA in dilute solution [5], by means of either measurements of solution properties [6–10] or atomistic molecular dynamics simulation [11] (particularly noteworthy are the pioneering works of Harvey, McCammon and coworkers [12–15]). Recent works and reviews on tRNA dynamics are those of Alexander et al. [16] and Agirrezabala and Valle [17]. Ribosomal RNAs are structurally quite more complex than tRNAs. For example, the secondary structure of the majority of the 5S rRNAs has five double helices, two hairpin loops, two internal loops, and a hinge region organized into three big domains (*α*, *β*, and *γ*) [18–20] as sketched in Fig. 1b. Combining results from dilute solution experiments with numerical calculations, some models for the tertiary structure of 5S rRNA have been proposed [19, 20].

The level of detail, or coarse-graining, of a macromolecular model depends on the type of behavior that is intended to capture. Thus, a large variety of coarse-grained models and computational methods are used for biological macromolecules [21]. Since long, our group has developed procedures and devised computational tools to build coarse-grained models and predict solution properties of both rigid [22–24] and flexible macromolecules [25–27]. The latter are represented by bead-and-spring chains where, in the classical concept, each bead corresponds to a large piece of the macromolecular chain. Nonetheless, finer coarse-grained (bead-and-spring) models can be used whose elements represent, for instance, the repeating units – amino acid or nucleotide residues – of the biomacromolecules [28, 29]. Indeed, one of the present authors proposed forty years ago [30] to obtain hydrodynamic properties for double-helical DNA using what is nowadays regarded as a coarse-grained model with one bead per nucleotide and the approximate Kirkwood formula for friction coefficients. The problem was revisited, in further work [28], using modern bead-model hydrodynamics [22] for rigid DNA models. Subsequently, bending and twisting internal flexibility was included in the model by means of stiff, elastic springs [31]. The latter simulation of flexible, double-helical DNA, which is precursor of the present work, is in the spirit of a popular family of coarse-grained models for biomacromolecules and supramacromolecular structures, the so-called “elastic network model” [32], which has been applied to RNA structures [33, 34].

Certainly, the preferred simulation method for short time scales has been, following the above mentioned pioneering works [11, 12, 15], the classical molecular dynamics technique. There are many commercial and public domain tools that implement that technique [21] which has been applied to a variety of RNA structures [35–37]. However, the large size of the macromolecules and the typical long time scales of their global dynamics are not usually accessible to conventional (atomic-level) molecular dynamics simulations. An alternative is to apply the Brownian dynamics simulation technique to some type of coarse-grained model. Thus, in this work, we have attempted to devise a scheme for predicting global solution properties, that would also mimic the internal dynamics, of RNAs using the simple coarse-grained model with one bead per nucleotide, with intra-molecular interactions represented by a minimalist elastic network, along with the Brownian dynamics technique.

## Methods

### Coarse-grained model

The RNA molecules (tRNA and rRNA) were modeled by bead-and-spring chains with one bead per nucleotide. In that model, nucleotides along the RNA strand form a string of touching beads all of them with the same hydrodynamic radius, *σ*=3.15 Å. That value is determined according to the features of the double-helical regions which are modeled following previous works for double-stranded DNA [28, 31], taking into account that RNA double helices appear usually in the A form whereas DNA is commonly found in B (or Z) form. The A form is a right handed double helix with the following features [38]: major groove width 3.8 Å, minor groove width 10.9 Å, helix radius 10 Å, rise 2.8 Å, inclination 16.1°, number of base pairs per helical pitch 11. On the other hand, the phase angle, *ϕ*, which define the gap between the complementary helices and therefore the shape and size of the grooves [28], was set to *ϕ*= 200°.

Beads are the model elements where the friction and the intra-molecular interactions take place. There are two types of intra-molecular interactions in the model: elastic interactions (represented by Hooke springs) and excluded volume interactions. Hooke springs are used to keep the connectivity between beads along the strand as well as the secondary structure in the helical regions. Also, they introduce the required degree of flexibility in the model [25] and form a kind of elastic network (see Fig. 2). For the sake of minimizing the amount of interactions but keeping the double-helical shape and the stiffness at short scale, we found it adequate [28] to connect each bead *i* (within every double-helical region) to:

1. its first neighbors along its helix piece (beads *i*±1), which keeps connectivity and bond equilibrium length.

**Fig. 2:**
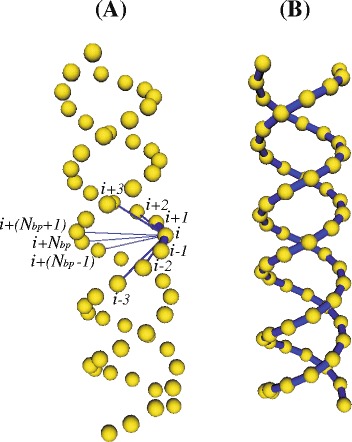
Double-helical model for RNA. **a** All the connectors supported by a given bead *i* (beads connected to *i* are labeled using *i* as reference). **b** All the connectors between first neighbors beads along each helix piece (i.e. connectors between each bead *i* and beads *i*±1). Beads appear smaller than in the real model (where they are tangent) for the sake of a better visualization of the connectors
2. its second neighbors along its helix piece (beads *i*±2), which accounts for bending interactions.
3. its third neighbors along its helix piece (beads *i*±3), which accounts for torsional interactions.
4. its counterpart in the complementary helix piece (bead *i*+*N*_*bp*_), which accounts for interactions between nucleotides forming the base pair.
5. the first neighbors of its counterpart in the complementary helix piece (beads *i*+(*N*_*bp*_±1)), which is necessary in order to keep the double helical structure.

*N*_*bp*_ is the number of base pairs within a given double-helical region. In the above description, we use the term “helix piece” instead of “strand” because every nucleotide in the RNA molecule actually belongs to a unique strand. Figure 2a shows the model for a double helix (in the straight, equilibrium conformation) displaying all the connectors involving one of the innermost beads (the rest of connectors was removed for the sake of clarity). On the other hand, each bead in a loop is only connected to their two first neighbors (beads *i*±1).

The Hooke spring potential reads

(1) $$ V_{ij}^{(conn)} = \frac{1}{2}H(l-l_{e}),   $$

where *l* is the instantaneous spring length, *l*_*e*_ is the equilibrium spring length, and *H* is the spring constant. Following previous works [31], we set $H=200k_{B}T/l_{e,1}^{2}$ for all connectors regardless the type of interaction represented. In that expression, *k*_*B*_*T* is the Boltzmann factor and *l*_*e*,1_ is the equilibrium length of the connector binding bead *i* to its first neighbor (*l*_*e*,1_=2*σ*=6.30 Å, in our touching-beads model). For the chosen value of *H*, the root-mean-square fluctuation in the spring length, $\sqrt {\langle l^{2} \rangle - \langle {l_{e}^{2}} \rangle }$, is 5 % of *l*_*e*_.

In addition to the elastic interactions, non-connected beads interact with each other through the following excluded volume (EV) Lennard-Jones potential that prevents beads from overlap and mimics good solvent conditions:

(2) $$ V_{ij}^{(LJ)}=4\epsilon_{LJ} \left[\left(\frac{\sigma_{LJ}}{r_{ij}}\right)^{12}-\left(\frac{\sigma_{LJ}}{r_{ij}}\right)^{6}\right],   $$

where *r*_*ij*_ is the instantaneous distance between the interacting beads, *ε*_*LJ*_=0.1*k*_*B*_*T*, and *σ*_*LJ*_=0.8*l*_*e*,1_ [39].

### Simulation, hydrodynamics, and solution properties

We performed Brownian dynamics (BD) simulations of bead-and-spring models of tRNA and 5S rRNA in order to obtain their solution properties in a solvent with viscosity *η*_*s*_=0.01 poise at *T*=293 K. The deterministic forces in the BD algorithm are the derivatives of the potentials defined by Eqs. (1) and (2). Both simulation and analysis were carried out with the software package SIMUFLEX [27] that implements a procedure based on the Ermak-McCammon algorithm [40]. That BD algorithm takes into account the hydrodynamic interaction (HI) effect through the Rotne-Prager-Yamakawa tensor [41]. Although the rigorous inclusion of the HI effect is necessary in order to generate the right macromolecular dynamics, BD simulations without HI (computationally less expensive) can be used as a “smart Monte Carlo” procedure [42] because they sample correctly the equilibrium conformational space. In such an approach, an ensemble of equilibrium conformations is generated and properties are calculated by means of the so-called rigid body treatment [43]. In the rigid body treatment, conformations are considered as instantaneously rigid and the hydrodynamic theory for rigid bead models is applied [23]. According to that procedure, conformational properties (like the radius of gyration) can be rigorously calculated whereas some overall hydrodynamic entities (like the translational diffusion and the intrinsic viscosity) can be acceptably estimated [44].

The initial conformations of the RNA models (including helices and loops) were built using an in-house program. After an equilibration period, long BD simulations with a duration about 100 times the relaxation time of the molecules were carried out. The resulting trajectories were divided into five pieces in order to calculate averages and standard deviations. We computed three properties: the radius of gyration *R*_*g*_, the translational diffusion coefficient *D*_*t*_, and the harmonic relaxation time *τ*_*h*_.

The macroscopic translational diffusion of a Brownian particle (consequence of its Brownian motion) is characterized by the time correlation function of the center of mass or Einstein equation 〈*d*^2^〉=6*D*_*t*_*Δt*, where 〈*d*^2^〉 is the mean squared displacement of the center of mass, *Δt* is the displacement duration, and *D*_*t*_ is the translational diffusion coefficient of the particle. Long BD-HI simulations were carried out to obtain *D*_*t*_ using that equation. On the other hand, BD-noHI simulations were used as a “smart Monte Carlo” procedure to generate equilibrium conformations and apply the rigid body treatment to obtain *τ*_*h*_ and *R*_*g*_. The harmonic relaxation time *τ*_*h*_ characterizes the overall tumbling of the molecule and can be compared to experimental rotational relaxation times. In case of a rigid body with arbitrary shape, the harmonic mean of its five characteristic rotational relaxation times is calculated as *τ*_*h*_=1/(6*D*_*r*_) [23], where *D*_*r*_ is a rotational diffusion coefficient obtained from the eigenvalues of the rotational diffusion tensor. Although the rigid body hydrodynamics is not strictly applicable to the reorientational dynamics of flexible structures (like tRNA and rRNA), it allows for the calculation of an ensemble average 〈*D*_*r*_〉 from which to calculate a value *τ*_*h*_=1/(6〈*D*_*r*_〉). Finally, since *R*_*g*_ is a conformational property, it is correctly obtained from a conformational average over a BD-noHI trajectory.

## Results and discussion

**Fig. 3:**
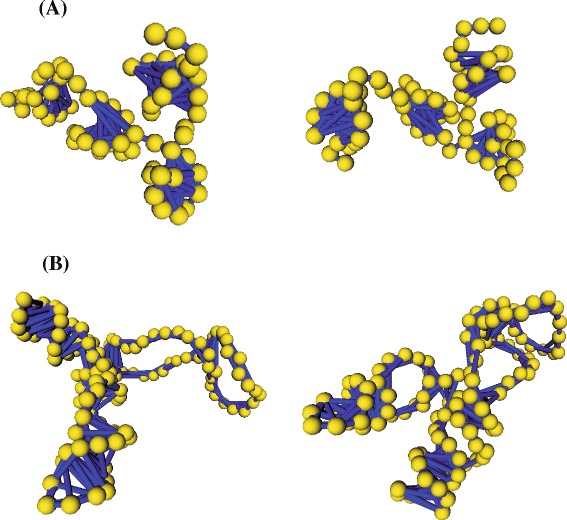
tRNA and rRNA models. Conformations along the Brownian trajectory (after equilibration) for **a** the tRNA model, and **b** the 5S rRNA model

**Fig. 4:**
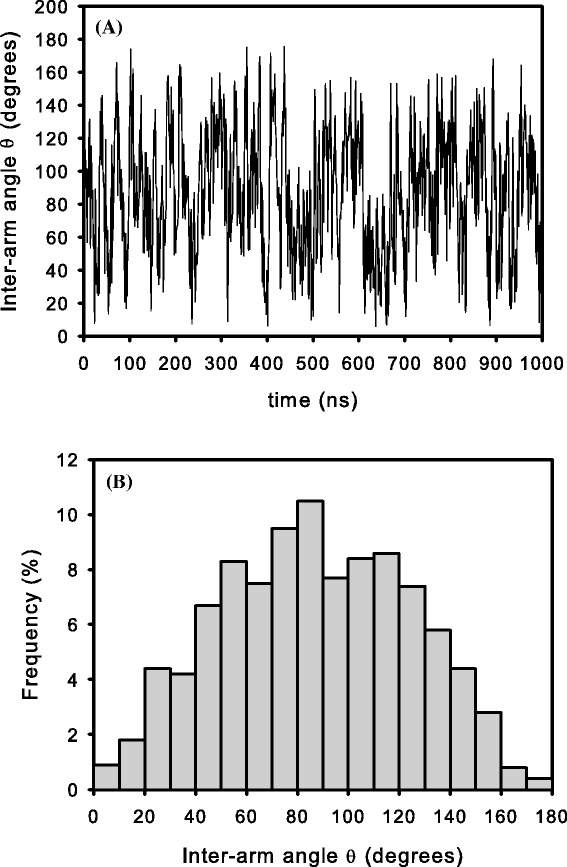
Inter-arm angle (*θ*). Angle subtended between the “acceptor stem” and the “aticodon stem” of tRNA: **a** fluctuation of *θ* along the Brownian trajectory; **b** frequency of occurrence of the different *θ* values

A bead-and-spring model for the yeast phenylalanine tRNA, yeast tRNA ^phe^ (PDB code 1EHZ), was built according to the structure sketched in Fig. 1a [45]. After a BD-noHI simulation for equilibrating the initial conformation, BD simulations with and without HI were performed. Figure 3a shows two of the many conformations adopted by that molecule along the Brownian trajectory once the equilibrium has been reached (the “Additional file 1” in the supplementary material displays the movie of the trajectory). Flexibility is noticed in the variation of the angle between arms (inter-arm angle *θ*) as better appreciated in Fig. 4a. There, it is depicted the fluctuation along the Brownian trajectory of the angle *θ* subtended by the two arm vectors, one defined along the “acceptor stem” and the other one defined along the “anticodon stem”. As observed, fluctuations are quick and the value of the angle varies in a relatively wide range, in agreement with some molecular dynamics simulations [11]. Figure 4b is an histogram that shows the frequency of occurrence of the different *θ* values as taken at intervals of 10°. Its broadness is indicative of the flexibility of the structure. Clearly, there is a most probable equilibrium value around *θ*∼ 90° (the precise average value being *θ*= 88°), which is coincident with that found in the crystal structure. Therefore, overall (time-averaged) hydrodynamic properties can be reasonably estimated using rigid bead models built upon the atomic coordinates coming from PDB files [46, 47].

Table 1 collects our BD results for *R*_*g*_, *D*_*t*_ and *τ*_*h*_, along with values calculated previously using rigid body hydrodynamics [47] and experimental results. The rigid body hydrodynamic calculations were carried out with the program HYDROPRO [24] which is able to build a bead model from the atomic (PDB) coordinates. There is a general good agreement among HYDROPRO calculations, BD simulations and experimental results. The advantage of our BD scheme is the possibility of evaluating, in addition to global solution properties, the internal dynamics of the molecule as illustrated by Fig. 4.

**Table 1:** Experimental and calculated values (via rigid body hydrodynamics and BD simulations) of solution properties of yeast tRNA ^phe^

Property	Experimental	Rigid body	Brownian
		hydrodynamics^a^	dynamics
*D*_*t*_×10^7^ (cm ^2^/s)	7.3 [45]; 7.9 [6]	7.89 [47]	7.8±0.5
*τ*_*h*_ (ns)	23.4 [7]	24.2 [47]	23.6±0.8
*R*_*g*_ (Å)	23.1 [7]	24.1 [47]	25.1±0.3

^a^Calculated from PDB file 1EHZ

On the other hand, a bead-and-spring model of 5S rRNA of *E. Coli* (PDB code IC2X:C) was built according to the structure sketched in Fig. 1b [20]. Again, after equilibrating the initial conformation, BD simulations with and without HI were performed. Figure 3b illustrates two of the many conformations adopted by our 5S rRNA model along the equilibrium Brownian trajectory (the “Additional file 2” in the supplementary material displays the movie of the trajectory). The flexibility is revealed by the variation of the angle between the domains (*α*, *β*, and *γ*) as well as the expansion of the loops whereas the helical regions keep a rigid structure.

Table 2 shows the values for *R*_*g*_, *D*_*t*_ and *τ*_*h*_ coming from our BD simulations along with some values selected from Skibinska et al. [20]. In that work, authors collect experimental data from several sources and present their own experimental data. Also, they make some rigid body hydrodynamic calculations (partly carried out with the program HYDROPRO [24]) and propose a likely tertiary structure for the 5S rRNA.

**Table 2:** Experimental and calculated values (via rigid body hydrodynamics and BD simulations) of solution properties of 5S rRNA of *E. Coli*

Property	Experimental	Rigid body	Brownian
		hydrodynamics^a^	dynamics
*D*_*t*_×10^7^ (cm ^2^/s)	6.00 [20]; 6.2 [48]	6.16 [20]	6.2±0.3
*τ*_*h*_ (ns)	107 [20]	79.25 [20]	82±2
*R*_*g*_ (Å)	32.7 [48]; 39 [49]	32.98 [20]	39.2±1.5

^a^Calculated from PDB file IC2X:C

As observed, the results coming from our BD simulations agree relatively well with both experimental data and rigid body hydrodynamic predictions. In particular, our predictions for *D*_*t*_ and *R*_*g*_ are coincident with some of the experimental measurements collected in Table 2 [48, 49]. If we use our predicted *D*_*t*_ value (6.2×10^−7^ cm ^2^/s) and the Svedberg equation ($s/D_{t}=M(1-\bar {v}\rho)/RT$), we obtain a value for the sedimentation coefficient *s*≃5 S what further supports the validity of the scheme employed in this work. In order to obtain that value of *s*, we assume for the 5S rRNA a molecular weight *M*=40000 Da [50] and a partial specific volume $\bar {v}=0.53$ cm ^3^/g [51] as well as a solvent (water) density *ρ*≃1 g/cm ^3^.

On the other hand our calculated *τ*_*h*_ is smaller than the experimental value obtained by Skibinska et al. [20], although close to the value predicted by their rigid body hydrodynamic calculations. As already mentioned, the rigid body hydrodynamic treatment does not capture rigorously the reorientational dynamics of flexible molecules and it is not the most adequate procedure to calculate their rotational relaxation times. For the case of 5S rRNA, the rigid body treatment seems to underestimate the rotational relaxation time. In any case, theoretical predictions are reasonably good if one considers the existing discrepancy among the experimental data themselves. In the “Additional file 1” (yeast tRNA ^phe^) and the “Additional file 2” (5S rRNA) of the supplementary material, one can appreciate the internal and rotational dynamics of the RNA molecules modeled in this work.

## Conclusions

We have devised a computational scheme to model RNA molecules, which present a partially disordered structure, and calculate their solution properties. According to the model, each nucleotide is replaced by a bead so that the secondary structure is represented in detail. The beads are connected by Hooke springs that form a type of elastic network. The model dynamics can be simulated by any Brownian dynamics algorithm. The algorithm and the analysis procedures employed in this work are implemented in our public-domain software SIMUFLEX available at http://leonardo.inf.um.es/macromol. The previously mentioned program HYDROPRO is also available at that web site.

The good agreement of our simulation results with some experimental data as well as with other theoretical results obtained by alternative numerical approaches ensures the validity of our scheme for the prediction of solution properties of RNAs. It is also indicative that the model is able to capture the tertiary folds of RNA molecules in solution. The advantage of the Brownian dynamics simulation technique (including hydrodynamic interaction) respect to other procedures is the possibility of simulating the long time internal dynamics of big macromolecules in real time.

In a previous work [52], we tested the adequacy of a computational scheme based on a residue level model and the Brownian dynamics simulation to predict the internal dynamics of partially disordered proteins. The present work on the more complex RNA molecule further confirms the success of that type of approach which is expected to be appropriate for the study of other disordered biological macromolecules.

## Availability of supporting data

All the supporting data are included as additional files.

## Additional files

Additional file 1
**Movie of the BD-HI equilibrium trajectory of our yeast tRNA** ^**phe**^ **model.** (AVI 26726 kb)

Additional file 2
**Movie of the BD-HI trajectory of our 5S rRNA model from the initial conformation.** (AVI 32972 kb)
